# Circulating CD34+ hematopoietic stem/progenitor cells paralleled with level of viremia in patients chronically infected with hepatitis B virus

**DOI:** 10.1051/rmr/170005

**Published:** 2018-02-20

**Authors:** Hussein Abdellatif

**Affiliations:** 1 Anatomy and Embryology Department, Faculty of Medicine, University of Mansoura, Mansoura Egypt; 2 Department of Anatomy, College of Medicine, University of Bisha, Bisha Saudi Arabia

**Keywords:** CD34, chronic liver injury, hematopoietic stem cells, hepatitis B virus, liver regeneration

## Abstract

**Introduction:** Liver regeneration is a heterogeneous process involving proliferation of different cell types in response to injury. Bone marrow derived stem cells may be involved in this process, by making contribution to parenchymal restoration and cellular replacement. We aimed to investigate the correlation between level of circulating mobilized CD34+ hematopoietic stem progenitor cells (HSPCs) and viremia level in patients chronically infected with hepatitis B virus (HBV).

**Methods:** Blood samples were prospectively collected for assessing percentage and absolute counts of circulating CD34+ HSPCs and viral load level using flow cytometry and RT-PCR respectively. Patients with chronic hepatitis B (CHB) (*n* = 30), Entecavir (ETV) treated subjects (*n* = 30) and 20 age and gender matched healthy controls were enrolled in this study. Results were expressed as mean ± SD.

**Results and discussion:** A significant increase in circulating CD34+ HSPCs level was observed in CHB patients (5 ± 3.1, 324 ± 195 × 10^3^/ml) as compared to ETV treated subjects (0.57 ± 0.27,1022 ± 325) and healthy controls (0.53 ± 0.37, 694 ± 254, *P* < 0.001) in regards to percentage and absolute counts respectively. Levels of CD34+ HSPCs strongly and positively correlated with HBV DNA viral load levels in CHB patients (*r*^2^ = 0.8417, 0.649, *P* < 0.001).Thus, in chronic liver disorders (CHB), when reduced regenerative capacity of hepatocytes is reached, BMSCs mobilization occurs and their level increases in peripheral blood. The level of circulating CD34+ cells in peripheral blood of CHB patients paralleled with the hepatitis B viral load.

## 1 Introduction

Hepatitis B virus (HBV) causes transient and chronic liver infections. Transient infections may produce serious illness, and approximately 0.5% ends with fatal, fulminant hepatitis. Chronic infections may also have major consequences: nearly 25% terminate in untreatable liver cancer [2]. Worldwide deaths from liver carcinoma caused by HBV infection is probably beyond one million per year [8,20]. Attempts to treat chronic infections have had some limitations in the past [12]. One of the reasons for chronic HBV infections is that the virus causes chronic, non-cytocidal infections of hepatocytes, the principal cells of the liver. Hepatocytes permanently shed virus into the bloodstream, ensuring that 100% of the hepatocyte population is infected. The combination of a long-lived non dividing host cell and a stable virus-host cell interaction virtually ensures the persistence of an infection in the absence of a robust host immune response [19].

In response to liver injury, liver regeneration is a heterogeneous process involving proliferation of multiple cell types [9,23]. Mature hepatocytes are considered the primary source for liver renewal and cellular replacement. When proliferation of hepatocytes is inhibited during extensive and chronic liver disease, a resident intrahepatic compartment of progenitor cells (HPCs) is activated. When both, mature hepatocytes and HPCs are exhausted and insufficient, bone marrow (BM)-derived hematopoietic stem cells (HSCs) may represent a third proliferative compartment. Both animal and human experiments revealed that transplanted BM stem cells can enter the liver from the circulation and give rise to hepatocytes and cholangiocytes [15,30,31]. The biological significance of such cells for liver repair and the situations of liver damage when they are actively recruited from the BM remain unclear. Acute and chronic liver injuries with different forms of cellular damage induces recruitment of these cells from the BM and their involvement in liver repair. However, the degree of their increase has not been demonstrated in previous studies [5]. In this regard our present study aimed to elucidate the correlation between level of circulating mobilized CD34+ HSCs and level of viremia in patients chronically infected with HBV.

## 2 Materials and methods

### 2.1 Patients and control groups

Chronic hepatitis B (CHB) patients, 40 patients displayed clinical, biochemical and virological evidence of CHB infections were included in this study. All patients were chosen according to the diagnostic criteria published before on hepatitis B infection [33] (HBs Ag positive for at least 6 months, HBe Ag and anti HBe Ab (positive or negative) and HBV DNA positive). Age range from 21 to 65 years for both male and female subjects (pregnant females were not included). Ability to provide informed consent indicating awareness of the investigational nature of this study was included as criterion of selection as well. After excluding 10 patients due to presence of co-infection or refusing to participate, blood sampling was performed in totally 30 consecutive patients. They were enrolled from February to December 2016, at hepatitis clinic, Liver Hospital, Mansoura, Egypt. Blood samples were taken at admission.

CHB treated group: 30 patients treated with entecavir (ETV) were included in this study. Standards for enrolling the ETV treated group required all patients to have continuously used ETV for more than 72 weeks, biochemical, virological and serological assessments as well as CD34+ HSPCs measurement of these patients were carried out at end of therapy.

For all patients and treated groups, selected consecutively in this research, there was no evidence of concurrent hepatitis C virus (HCV), hepatitis D virus (HDV), hepatitis E virus (HEV) or human immunodeficiency virus (HIV) infection, as well as no evidence of autoimmune liver disease, no hepatocellular carcinoma or metastatic liver tumor, and no immunosuppressive medication at the whole period of the study. Full baseline characteristics and virological data of enrolled subjects are summarized in Table 1.

**Table 1:** Clinical characteristics of the enrolled subjects.

Parameters	CHB (*n* = 30)	CHB with ETV (*n* = 30)	Control (*n* = 20)	*P* value
Male/Female	20/10	18/12	14/6	
Age (year)	45 ± 8.2	45 ± 12.7	43 ± 8.2	0.351
ALT (IU/L)	237 ± 96.8*	20 ± 4.2	18.3 ± 3.8	<0.0001
Total bilirubin (mg/dL)	1.75 ± 0.9*	0.7 ± 0.32	0.6 ± 0.3	<0.0001
INR	1.1 ± 0.2	1,12 ± 0.23	1 ± 0.09	0.108
WBC count (×10^3^/uL)	6.8 ± 1.7	7.3 ± 2.3	6.9 ± 2	0.923
HBV DNA( log_10_ copies/mL) (P/N)	8.3 ± 4.5	<LOD (4/26)	ND	
HBsAg (positive/negative)	30/0	27/3	ND	
HBeAg (positive/negative)	6/24	7/23	ND	
Anti-HBe Ab (positive/negative)	24/6	23/7	ND	
Prior medication for HBV	0	ETV	0	

CHB = Chronic hepatitis B; ETV = Entecavir; ALT = Alanine aminotransferase; INR = International normalized ratio; WBC = White blood cells; LOD = Limit of detection (20 IU/mL); P = Positive; N = Negative; ND = Not determined. Data are expressed as mean ± SD.

* Significant deviation from the control as indicated by *P* value < 0.05.

Healthy controls, Peripheral blood from 20 age and sex-matched healthy donors were used as controls to determine the normal values of circulating CD34+ cells. All subjects provided written informed consent before entering the study protocol and the study protocol was approved by local ethical committee at liver hospital, Mansoura, Egypt.

### 2.2 Virological assessments

Serum HBV DNA load was assessed with real-time polymerase chain reaction (Real-Time-PCR), quantitative fluorescent method, using a Light cycler PCR system (COBAS^®^ TaqMan^®^ 48 Analyzer, Roche, Branchburg, NJ) with a lowest limit of detection (LOD) of approximately 20 IU/ml. The experimental procedures were performed in strict accordance with the reagent kit (COBAS^®^ TaqMan^®^ HBV test, CAP-CTM; Roche Molecular Systems, Inc., Branchburg, NJ) package insert. The primer was provided in the kit, the reaction volume was 60 µL, and the reaction condition was 37 °C for 5 min, 94°C for 1 min then 40 cycles as 95 °C for 5 s and 60 °C for 30 s. HBV markers (HBsAg, HBsAb, HBeAg, anti-HBeAb) were measured by ELISA (enzyme-linked immunosorbent assay) method. The experimental methods followed the guidelines written in the reagent kit (R&D, MN, USA).

### 2.3 Flow cytometric analysis

Blood samples were obtained in the outpatient department for the study groups and also for the healthy control subjects. Ten milliliters of blood was drawn from the antecubital vein. Mononuclear cells (MNCs) were then isolated by density-gradient centrifugation of Ficoll 400 (Ficoll^®^ Paque Plus, GE17-1440-02, Sigma-Aldrich, Germany) as previously described [3]. The isolated MNCs were washed twice with phosphate buffer solution (PBS) and centrifuged before incubation with 1 mL blocking buffer (BSA) for 30 min at 4 °C. Cell viability of >95.0% was noted in each group. The phenotype of circulating cells was evaluated by conventional dual color immunofluorescence using PE-conjugated anti-CD34, vioblue-conjugated anti-CD45 (Miltenyi Biotec, Germany). Briefly, small aliquots of MNCs (4 × 10^5^) were incubated with 10 μL PE-conjugated anti-CD34 antibody and vioblue conjugated anti-CD45 for 15 min in the dark at 4 °C. PE or Vioblue conjugated anti-mouse IgG were used as isotype controls. Erythrocytes were lysed with lysis buffer (0.155 M NH_4_Cl, 0.012 M NaHCO_3_, 0.1 mM EDTA, pH 7.2) for 5 min at room temperature. After washing with PBS, the cells were fixed with 1% paraformaldehyde and then analyzed by flow cytometry (MACSQuant Analyser, 2440, Miltenyi Biotec, Germany). Data were analyzed using MACSQuantify software (version 2.4) following International Society of Hematotherapy and Graft engineering (ISHAGE) acquisition layouts [24]. The assays for HSPCs in each sample were performed in duplicate and the mean levels were reported. The results were expressed as percentage (%) of total MNCs per sample. The absolute counts of positive cells were calculated using the two-platform approach. CD34+ cell count in the sample is calculated by multiplying the percentage value by the absolute PBMCs number as provided by the hematology analyzer (CD34% × PBMNCs count/ul × Dilution factor), this approach allows the discrimination of HSPCs (which express relatively low levels of CD45 on their surface) from lymphocytes and monocytes, and thus allowing the verification of “true” CD34+ cells as being dim for CD45 fluorescence and having low side scatter (CD45 dim, SSC low) [25,26]. Intra-assay variability based on repeated measurement of the same blood sample was low with a mean coefficient of variance of 4.2% in CHB patients, 4.15 in CHB treated group and 4.1% in healthy control subjects.

### 2.4 Statistical analysis

The results were expressed as mean ±SD. Statistical significance was determined by a two-tailed student's t test or one way ANOVA adjusted for multiple comparisons with a *P* value less than 0.05 considered statistically significant. Associations between variables were assessed using Pearson rank correlations. Serum HBV DNA results which were below the lower LOD (<20 IU/mL) were analyzed as being 20 IU per milliliter for the ease of analysis. Data processing was carried out with SPSS, 17.0.Statistical package for windows.

## 3 Results

### 3.1 Clinical characteristics of the study populations

Table 1 shows the baseline characteristics and laboratory findings of CHB patients, ETV treated subjects and healthy control group. The age, INR and WBCs count did not show significant difference between study groups. However, serum total bilirubin (1.75 ± 0.9 mg/dl) and ALT levels (237.7 ± 96.8 IU/L) were remarkably elevated in CHB patients than those in ETV treated (0.7 ± 0.32, 20 ± 4.2) and control (0.6 ± 0.3, 18.3 ± 3.8) groups respectively.

Pair-wise comparison between ETV treated and control subjects revealed no significant difference in total bilirubin and serum ALT levels (*P* value > 0.05).

CHB diagnosis based on serum expression of HBs Ag, HBe Ag and anti HBe Ab. Mean level of serum HBV DNA load is presented as log copies/mL. Among all 30 patients enrolled in the study, HBV load was (8.4 ± 4.5 log copies/mL) which was significantly elevated when compared to ETV treated (Responders) and healthy control groups who were below the detection limit (20 IU/mL).

In ETV treated group, 26 subjects achieved significant viral suppression with their DNA mean level decreased to undeletable limit <20 IU/mL). However, 4 patients failed to respond to ETV therapy, either no virological improvement or with later rebound at study time.

**Fig. 3:**
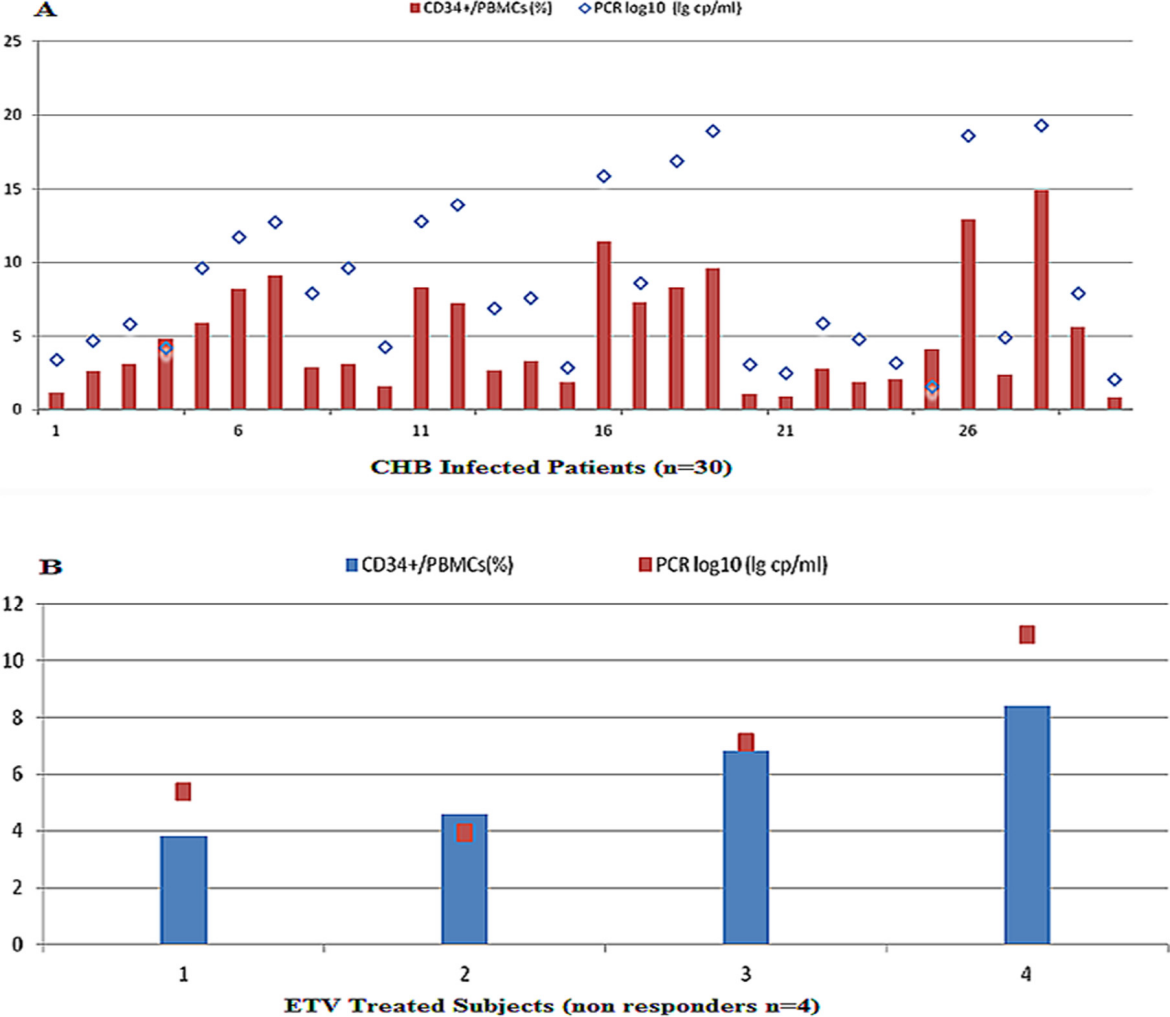
Changes in CD34+ HSPCs and hepatitis B virus load in peripheral blood of (A) CHB patients; (B) Entecavir (ETV) non responders. A Percentage of CD34+ cells in PBMCs fraction and HBV DNA load (log_10_ copies/mL) are expressed.

Changes in CD34+ circulating HSPCs were analyzed based on patients' virological responses (Fig. 3A and B).

### 3.2 Circulating CD34+ hematopoietic stem/progenitor cells in CHB patients and ETV treated groups

**Fig. 1:**
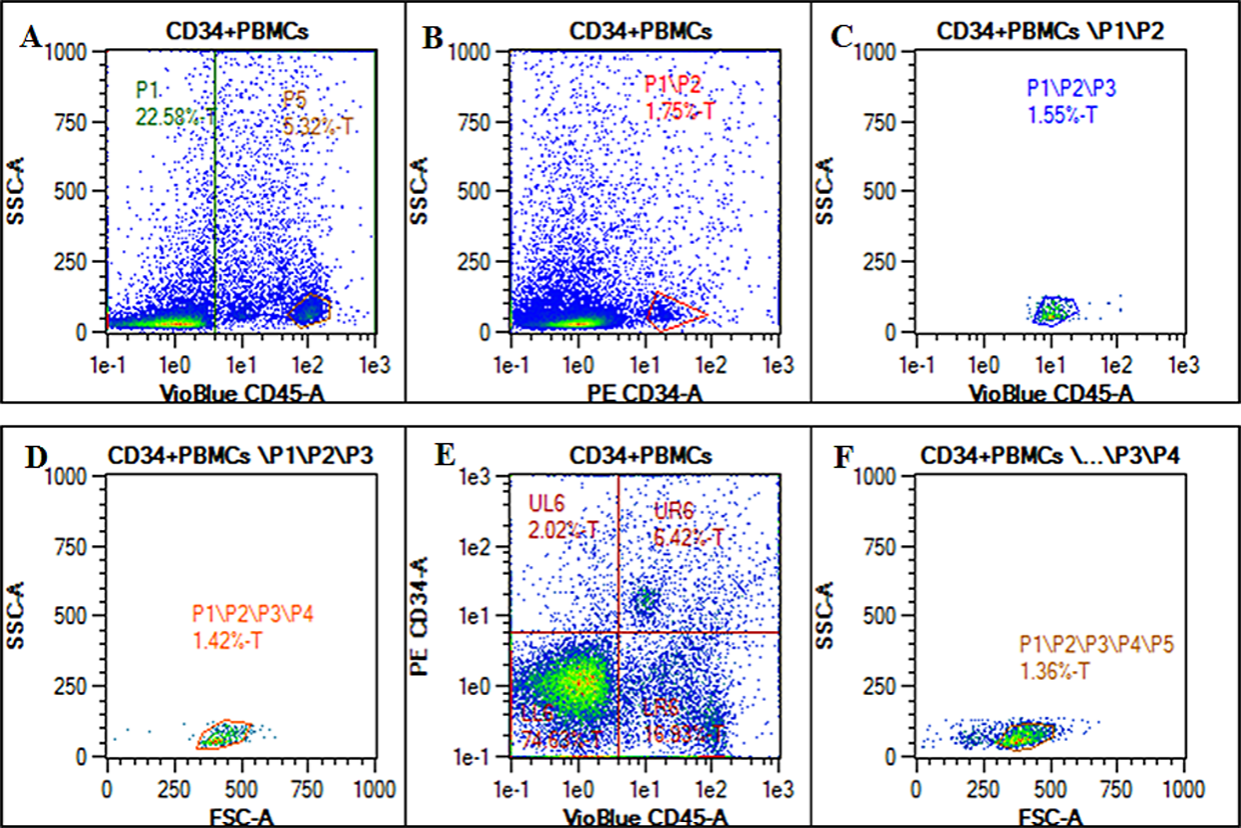
CD34+ hematopoietic stem progenitor cells detected in peripheral blood of CHB patients by flow cytometry using ISHAGE logical gating strategy. Small aliquots of PBMCs were incubated with PE-conjugated anti-CD34 antibody, vioblue-conjugated anti-CD45 and analyzed by flow cytometry. (A) representative plots of whole MNCs shown by CD45 vs. side scatter. The initial gate P1 is set to include all CD45+ events including CD45^dim^ and CD45^bright^; (B) the events in gate P1 are then displayed on a CD34 vs. SSC dot plot and a second gate (P2) produced to include the cluster of CD34+ events; (C) this plot is obtained by plotting the events that fulfill the criteria of gates P1 and P2 (sequential gating), Cells forming a cluster with characteristic low SSC and low to intermediate CD45 fluorescence are then gated on this third plot to produce a region (P3); (D) events fulfilling the criteria of all three gates (P1, P2 and P3) are then displayed on a FSC vs. SSC dot plot to confirm that selected events fall into a ‘lymph-blast’ region (P4); (E) ungated data are displayed on a CD34 vs. CD45 histogram to establish the lower limit of CD45 expression by the CD34+ events; (F) region (P5) is set to include events no smaller than lymphocytes by back scattering a small number of lymphocytes from plot 1 (high CD45 staining with low side-scatter). Any events falling outside region P5 are excluded from final calculation. PBMCs: peripheral blood mononuclear cells; T: total events acquired.

In an attempt to quantitatively analyze the level of circulating CD34+ HSPCs in the peripheral blood of patients, the aliquots of blood samples of 30 CHB patients, 30 ETV treated subjects and 20 healthy controls were assessed by dual color flow cytometry. MNCs were first gated by CD45 positive staining (Fig. 1A). Then cells were further gated by CD34 positive staining (Fig. 1B). A representative of double-positive cells (CD34+/CD45+) from one CHB patient is shown in Figure 1C–F.

#### 3.2.1 Percentage of circulating CD34+HSPCs

**Fig. 2:**
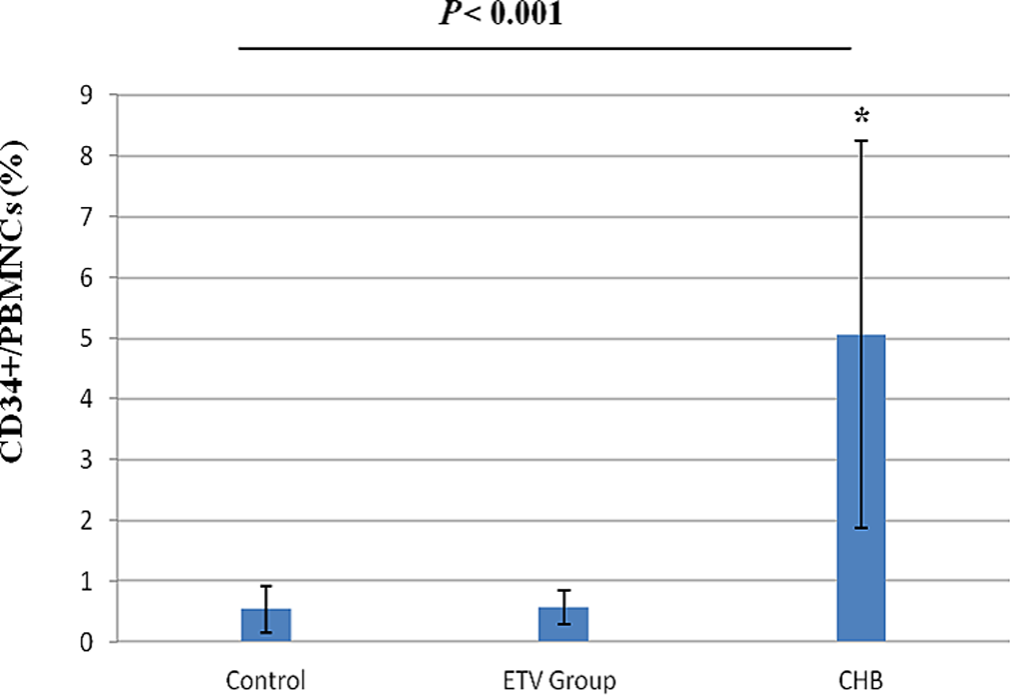
Elevation of CD34+ HSPCs in the peripheral blood of CHB patients. A Percentage of CD34+ cells in PBMCs fraction. Data represent the percentages of circulating CD34+ cells in the PBMCs fractions of 30 CHB patients, 30 ETV treated patients and 20 healthy controls. Values are expressed as mean ± SD. *indicates significant deviation from the control as indicated by *P* value < 0.05.

As shown in Figure 2, a baseline of the percentage of CD34+/CD45+ cells in the PBMCs ranged from 0.01 to 1.3% (0.53 ± 0.37%) in healthy controls. In comparison with healthy controls, a significant increase in the percentage of circulating CD34+ cells, which ranged from 0.8 to 14.9% (5 ± 3.1, *P* < 0.001), were observed in CHB patients.

In ETV treated patients (with good response), the percentages of CD34+ cells in PBMCs ranged from 0.2 to 1.1 (0.57 ± 0.27) which was significantly lower than that of CHB patients (*P* < 0.001), but with no difference in comparison to healthy controls. In contrast, patients with poor response to ETV (4 patients) showed increase of their CD34+ HSPCs level (5.9 ± 2.09) as compared to control level (*P* < 0.001).

#### 3.2.2 Absolute count of circulating CD34+HSPCs

The average absolute numbers of CD34+ HSPCs in peripheral blood MNCs of CHB infected patients was (324 ± 195.9 × 10^3^/mL), but only (694 ± 254) in healthy controls, indicating significant increase of CD34+ cells in CHB patients (*P* value < 0.001). However, in ETV treated patients (with good response), the CD34+ HSPCs were (1022 ± 325) which was significantly lower than that of CHB infected patients (*P* < 0.001) but with no significant difference when compared to healthy controls. In patients with poor response to ETV (4 subjects), increase in CD34+ cells level was evident (278 ± 132 × 10^3^/mL) when compared with healthy controls (*P* < 0.001) and there was no significant difference when compared to CH34+ HSPCs level in CHB infected patients (*P* value > 0.05).

#### 3.2.3 CD34+ HSPCs values followed that of HBV DNA level

**Fig. 4:**
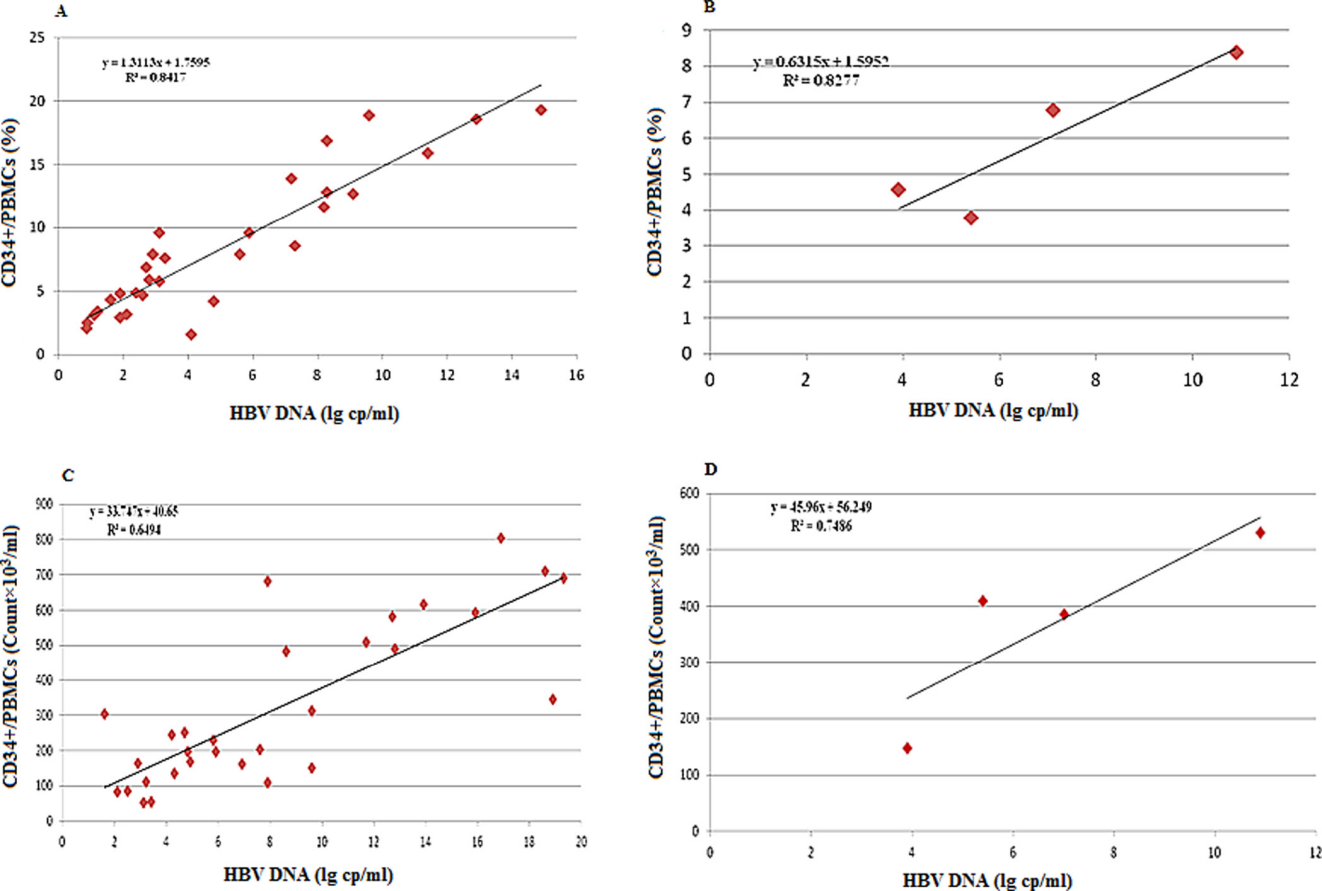
Correlation between CD34+ HSPCs (percentages (%) and absolute counts) in the peripheral blood and mean HBV DNA load in (A) CHB patients; (B) Entecavir (ETV) non responders (%); (C) CHB patients; (D) Entecavir (ETV) non responders (absolute counts). HBV: Hepatitis B virus; PBMCs: peripheral blood mononuclear cells. (*r*^2^ = 0.841 and 0,649 respectively, *P* < 0.001).

As it is apparent from Figure 3A and B, CD34+ HSPCs values followed that of HBV DNA level. Further analysis from all available data paired for viral load and percentage or absolute counts of CD34+ HSPCs subset indicated that circulating CD34+ HSC subpopulations in CHB patients are positively related to serum HBV DNA log values (Fig. 4A–D, *r*^2^ = 0.841 and 0.649 respectively, *P* < 0.001).

## 4 Discussion

The HBV infection is a global public health burden. Despite availability of effective vaccines since the 1980s, nearly one third of the world's population has serological evidence of past or incident infection with HBV [18]. Without effective treatment, CHB patients eventually develop liver cirrhosis or hepatocellular carcinoma. The complex trafficking of mature hepatocytes, intrahepatic and extrahepatic stem cells in presence of liver damage suggests that liver repopulation is a heterogeneous phenomenon driven by variant cell types and molecular mechanisms [1,5,6,7,21]. Accumulating evidence has revealed that the BM-derived human HSPCs can migrate to the liver and contribute to liver regeneration [14,27]. De Silvestro and his associates showed that in a series of 13 patients subjected to hepatectomy, CD34+ peripheral cells increased compared to both healthy controls and to patients submitted to abdominal surgery other than hepatectomy [4].

CHB is associated with massive cell death and consequently, impairment of liver functions (Table 1). So far, there is only limited data available on the dynamics of circulating BM-derived HSCs in CHB patients. In this study, we elucidated that CHB patients have more than nine-fold elevations of circulating CD34+ HSPCs in comparison with healthy controls and CHB patients treated with ETV. The circulating CD34+ cells co-expressed the hematopoietic marker CD45.

Wynn [32] stated that inflammation is a critical factor in the initiation and maintenance of liver fibrogenesis and cirrhosis from HBV infection. Inflammation reactions and release of inflammatory mediators from damaged cells, such as TGF-beta1 and TNF-alpha, can recruit reacting cells in circulating blood to the liver [32]. Thus, it seems that elevated CD34+ levels in peripheral blood of CHB patients are related to persistent liver inflammation (detected by elevated levels IL-8 and IFN-α in CHB patients') and imbalance between injury and endogenous repair capabilities. However, in ETV treated patients who achieved a good response, patients had similar level of CD34+ cells with healthy controls, suggesting that ETV mediated inhibition of HBV replication (reducing the HBV effect on cell mediated responses) can improve the liver environment and achieve balance between injury and endogenous regeneration. This may be critical for liver tissue regenerative process in which BM derived stem cells are actively involved. In contrast, patients with poor response to ETV therapy had elevated levels of CD34+ HSPCs (5.9 ± 2.09) when compared to healthy controls, these elevated levels may be related to virological failure and persistent liver inflammation with mobilization of HSCs from the BM. Interestingly, there was a strong correlation between viral hepatitis load and circulating CD34+ cells in CHB patients and ETV treated groups (Fig. 4A–D, *r*^2^ = 0.8417, *P* < 0.001). Previous reports have demonstrated the level of CD34+ HSCs and the different mobilizing factors leading to their increase in patients with HBV infection or in experimentally induced acute liver failure [16,29].

Our results appear to go with previously published literature in this field suggesting that the role of extrahepatic progenitors in liver repopulation is extremely limited in normal and transiently injured liver [11,13,28,30], but in chronic liver disorders, when reduced regenerative capacity of hepatocytes is reached, BMSCs mobilization occurs and their level increases in peripheral blood [17]. However, it appears to be contradictory to Di Campli et al. who stated that circulating CD34+ stem cells are not recruited in patients with a broad range of hepatic injuries [5]. The contradictory results can probably be explained by the small number of patients enrolled in their studies (8 patients) and variation may be related to HBV genotype difference among both population studies.

Possible correlation between viral load level and circulating CD34+ HSPCs in patients receiving anti-viral therapy also been investigated by Lv et al. [17]. They discussed the effect of nucleoside analogues (ETV) on CD34+ HSCs in CHB patients and detected increase in CD34+ HSCs in ETV treated subjects. Contradictory results may be related to use of CD34 only as a marker of peripheral and liver tissue cells. Other cells like circulating endothelail cells (cEC) express of CD34 as well, absence of CD45 expression is a hallmark difference between both cell types [10]. Different isolation techniques, enrichment prior to analysis and sequential gating strategy may be other contributing factors for variable results. Parallel correlation of hepatitis B viral load and circulating CD34+ HSPCs in patients receiving antiviral agents needs further exploration. This study reported that the circulating level of CD34+ HSPCs is significantly correlated with the viral load in CHB infected patients.

The study has limitations. First, the exact mobilizing factor that may be significantly involved in HSPCs' mobilization and/or liver repair is not clearly defined (Though ELISA detection of hepatocyte growth factor (HGF) and stem cell factor (SCF) was performed but their correlation with both viral load and CD34+ HSPCs was not detected), role of other mobilizing factors as SDF-1 alpha and G-CSF need to be explored and analyzed. Second, our study subjects belong to genotype D (most prevalent in Egypt) [22], the lack of other genotypes may have led to bias in patients' selection. Patients with other genotypes should be examined further for possible correlation of CD34+ cells with hepatitis B viral load. To minimize other selection bias, a clearly defined standard was used to select cases and control subjects and ensure that they are truly representative of the entire population. They were selected randomly from the outpatient clinic, subjects not fulfilling the selection criteria were excluded from the study. Finally, despite our interesting findings, the sample size of our series is relatively small. All conclusions based on our results are, therefore, tentative and need further support from well-controlled clinical studies of larger scale with wide range of study subjects.

## 5 Conclusion

The results of our study demonstrated that the “wear and tear” in chronic liver diseases (CHB) is associated with increased level of circulating CD34+ HSPCs. Level of such cells in peripheral blood of CHB patients is paralleled with the hepatitis B viral load. Our data suggest that future studies in humans should probably be extended to patients affected by other forms of hepatitis and other clinical situations to verify the possible mobilization of extrahepatic progenitors under critical conditions and to eventually confirm the association of viral load with the level of circulating cells and assess the malleability of these cells.

## Nomenclature

BMSCs: bone marrow stem cells
BSA: bovine serum albumin
cEC: circulating endothelial cells
CHB: chronic hepatitis B
ETV: entecavir
HBV: hepatitis B virus
HPCs: hepatic progenitor cells
HSPCs: hematopoietic stem progenitor cells
INR: international normalized ratio
IFN-α: interferon alpha
IL-8: interleukin-8
MNCs: mononuclear cells
SD: standard deviation

## Competing interests

The author declares that he has no competing interests related to this article.

## Declaration

### Ethics approval and consent to participate

All subjects provided written informed consent before entering the study protocol. Principles of blood sampling were followed in all experimental protocols and study protocol was approved by local ethical committee in liver hospital, Mansoura, Egypt.

### Availability of data and materials

The datasets during and/or analysed during the current study available from the corresponding author on reasonable request (Based on journal copyright transfer agreement).

## Funding

This research received no specific grant from any funding agency in the public, commercial, or not for profit sectors.
